# Prevention of depression and sleep disturbances in elderly with memory-problems by activation of the biological clock with light - a randomized clinical trial

**DOI:** 10.1186/1745-6215-11-19

**Published:** 2010-02-23

**Authors:** Els IS Most, Philip Scheltens, Eus JW Van Someren

**Affiliations:** 1 Netherlands Institute for Neuroscience, an Institute of the Royal Netherlands Academy of Arts and Sciences, Meibergdreef 47, 1105 BA Amsterdam, the Netherlands; 2 Department of Neurology, VU University Medical Center, De Boelelaan 1117, 1081 HV Amsterdam, the Netherlands; 3 Department of Integrative Neurophysiology, VU University Amsterdam, De Boelelaan 1085, 1081 HV Amsterdam, The Netherlands; 4 Department of Neurology, Leiden University Medical Center, Room T5-32, PO Box 9600, 2300 RC Leiden, The Netherlands

## Abstract

**Background:**

Depression frequently occurs in the elderly and in patients suffering from dementia. Its cause is largely unknown, but several studies point to a possible contribution of circadian rhythm disturbances. Post-mortem studies on aging, dementia and depression show impaired functioning of the suprachiasmatic nucleus (SCN) which is thought to be involved in the increased prevalence of day-night rhythm perturbations in these conditions. Bright light enhances neuronal activity in the SCN. Bright light therapy has beneficial effects on rhythms and mood in institutionalized moderate to advanced demented elderly. In spite of the fact that this is a potentially safe and inexpensive treatment option, no previous clinical trial evaluated the use of long-term daily light therapy to prevent worsening of sleep-wake rhythms and depressive symptoms in early to moderately demented home-dwelling elderly.

**Methods/Design:**

This study investigates whether long-term daily bright light prevents worsening of sleep-wake rhythms and depressive symptoms in elderly people with memory complaints. Patients with early Alzheimer's Disease (AD), Mild Cognitive Impairment (MCI) and Subjective Memory Complaints (SMC), between the ages of 50 and 75, are included in a randomized double-blind placebo-controlled trial. For the duration of two years, patients are exposed to ~10,000 lux in the active condition or ~300 lux in the placebo condition, daily, for two half-hour sessions at fixed times in the morning and evening. Neuropsychological, behavioral, physiological and endocrine measures are assessed at baseline and follow-up every five to six months.

**Discussion:**

If bright light therapy attenuates the worsening of sleep-wake rhythms and depressive symptoms, it will provide a measure that is easy to implement in the homes of elderly people with memory complaints, to complement treatments with cholinesterase inhibitors, sleep medication or anti-depressants or as a stand-alone treatment.

**Trial registration:**

ISRCTN29863753

## Background

As the western population is increasingly aging, problems connected with old age will dominate health care. Depression, one of the most prevalent psychiatric disorders, is expected to take an even more prominent position than presently, as the risk for developing depression increases with old age. Depressive symptoms are present in almost one third of the elderly population and major depression may be present in up to 4% [1,2]. Furthermore, once present, the prognosis for elderly with depression is poor [3].

In 2007, an estimated 27 million people worldwide were diagnosed with Alzheimer's Disease (AD), the most common form of dementia, and this number is expected to quadruple by 2050 [4]. Although the most prominent symptom is cognitive decline, in time other distinctive symptoms develop, such as sleep-wake rhythm disturbances [5-10], behavioral disturbances including agitation and psychosis [11-15] and mood disturbances, notably depression [16,17].

### Depression in dementia

Depression prevalence can reach up to 18% in AD patients [18,19]. The occurrence of a major depressive episode in AD is as high as 20-25%, and minor depressive symptoms occur in an additional 20-30% [20,21]. Once they are present, depressive symptoms are persistent [3,22]. This shows that depression is common, especially in early/mild and moderate AD patients. Although Verkaik (2007) stated that the prevalence of depression between mild and severe dementia do not differ much [23], this is difficult to assess in severe dementia, something which may indicate an underestimation of the actual occurrences.

The consequences of depression can be considerable. Depression not only has a significant impact on the patient's quality of life and activities of daily life [16,17,24], it is also one of the major causes for depression in caregivers [25,26], all of the above contributing to early institutionalization of the patient. Additionally, the presence of mood disturbances is associated with increased mortality [27].

The etiology of depression in AD is still largely unknown. Being aware of having AD or the loss of (cognitive) function seems unrelated [28]. There is some evidence that the neuropathological features of AD play a role; Alzheimer pathology in the locus coeruleus [29] may be involved, as well as the dorsal raphe serotonergic nuclei [30]. However, newer studies have failed to replicate these data [31,32], and the precise part of AD neuropathology in the risk of developing depression is still an ongoing discussion.

A family history of depressive symptoms is the most prominent risk factor for developing depression in AD. A personal history of depression, gender and a young onset of AD also contribute significantly [33]. A key predictor of depressive symptoms is the presence of sleep disturbances [34-37]. Elderly experience more difficulties with sleeping than the young [38], increasing the probability of developing mood problems. As sleep problems become very distinctive in AD [5], it can be assumed that the chances of developing mood disorders are even higher.

### How light therapy might work

Light activates retinal ganglion cells which in turn excite the hypothalamic suprachiasmatic nucleus (SCN), the biological clock of the brain, via the retinohypothalamic tract. The SCN is the circadian pacemaker, generating and synchronizing biochemical and behavioural rhythms, for example, the regulation of melatonin via the paraventricular nucleus and cortisol through the hypothalamic-pituitary-adrenal axis [39]. Maintaining an endogenous rhythm of approximately 24.2 hours, the SCN is synchronized to the environmental day and night by light, to which it is extremely sensitive. A regular input is therefore necessary to ensure optimal coupling to the environmental 24-hour light-dark cycle. During the aging process, ocular light transmission may be impaired by age-related deficiencies in the eye (e.g., cataract) and degeneration of the optic nerve, creating a demand for higher light intensities in order to maintain a sufficient input to the SCN [8]. Without this increase, the SCN input gradually diminishes, which may be involved in the finding that SCN neurons show decreased activation at high age, and even more so in demented elderly [40]. These changes are likely to affect the pacemaker function of the SCN, showing up as arrhythmia in the sleep-wake cycle. Indeed, the severity of sleep-wake rhythm disturbances is strongly correlated with the loss of vasopressin neurons in the SCN [41].

Combined with the neuropathological effects of AD, decreasing SCN responsiveness even further due to increased cell(pathway) loss [42], previous studies suggest that it is possible to reactivate the SCN and improve sleep-wake rhythms by applying extra light, as will be summarized briefly in the next paragraph.

### Bright light therapy in AD

Since the early 1990s several researchers have evaluated the effect of bright light in demented elderly. Frequently reported positive effects are improvements of disturbed sleep and activity rhythms [12,43-48]. However, some studies fail to show these effects [49-51].

Only a handful of researchers have studied the effects of light therapy on mood in AD. Whereas efficacy could not be demonstrated in short-term studies with either three weeks of dusk and dawn simulation [52] or four weeks of one hour of morning light [12], Dowling et al did find small positive effects after ten weeks of one hour of morning light [53]. Even more pronounced effects on mood were found with long-term (up to 3,5 years) of whole-day bright light [43]. Although these findings concertedly suggest that effects may take weeks to develop, the best methodological approach to light therapy in AD is still unknown. Also, only a limited number of the studies on light therapy in dementia classify as randomized clinical trials. Furthermore, of all light therapy studies, the study of Riemersma et al (2008) has been the only one investigating long-term effects (up to 3,5 years). Finally, only a few studies investigated effects in community-dwelling patients [49]. Thus, additional randomized clinical trials are warranted, especially for investigating effects of long-term treatment on mood in community-dwelling elderly with memory complaints, either diagnosed with early Alzheimer's disease or with conditions that may develop into Alzheimer's disease.

### Advantages of light therapy

Light therapy provides a safe treatment option, with only mild side effects [54]. One study even reported a decrease in the frequency of common health complaints with bright light [43]. In non-demented people, light therapy is an accepted and validated treatment for seasonal affective disorder, but has also been reported to be effective in non-seasonal depression [55]. Acceptance among patients is high and it is a feasible option for patients who do not tolerate or are opposed to medication, or when medication is not effective [56].

### Ocular safety

The light intensity used is well within the range of normal daylight exposure. Furthermore, the filters applied on the light devices reduce the ocular UV and blue light hazard to far below the risks of exposure to natural daylight. Although not specifically examined in dementia patients, its long- term safety has been demonstrated in seasonal affective disorder patients by ophthalmologic examinations after exposures of up to 1250 hours [57].

Given these considerations, the aims of the current study are to investigate whether long-term daily bright light exposure is an effective treatment in home-dwelling elderly with memory problems who are at an increased risk for the development or worsening disturbed mood and sleep.

## Methods and design

### General objectives

The purpose of this study is to investigate the following primary hypothesis:

1. Long-term daily bright light exposure attenuates the occurrence of depressive symptoms.

Secondary hypotheses are:

1. Long-term daily bright light exposure attenuates the occurrence of sleep-wake rhythm disturbances.

2. Long-term daily bright light exposure ameliorates cognitive deficits.

3. Long-term daily bright light exposure ameliorates caregiver burden.

4. The effects of light on mood and cognition are in part mediated by its effect on the circadian pacemaker, as read out from the 24-hour rhythms in activity, body temperature, heart rate and cortisol.

### Procedure

**Figure 1:**
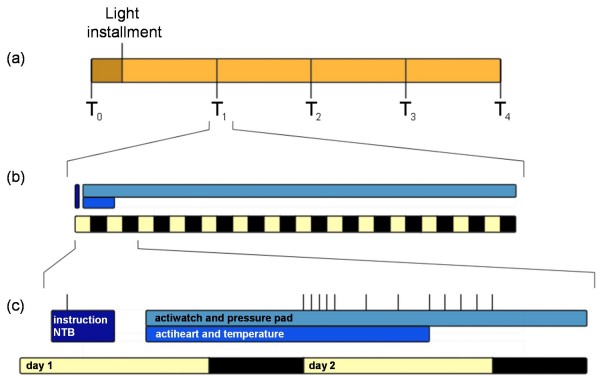
**Design and protocol**. After T_0_, patients receive a light box at home, where after they complete a maximum of four follow up visits, T_1_-T_4_, every 6 months (a). Each visit comprises the patient coming to the VU University medical centre, to receive instructions, perform a NTB, and practice the saliva sampling. At home, actigraphy is measured for two weeks, and temperature and heart rate are measured for 24 hours (b). Saliva sampling occurs on day 2 (timing indicated by vertical lines) (c).

After a baseline measurement (T_0_), all patients receive either a placebo or an active light box (see *interventions *and *randomization*). They are then followed-up for a maximum of four half yearly visits (T_1_, T_2_, T_3 _and T_4_) (figure 1, Design and protocol). For comparison, a baseline assessment is obtained in healthy controls at T_0 _only.

In short, at all assessments (T_0 _to T_4_) patients visit the VU University medical center for two hours, which start with an explanation of the procedures and administration of the questionnaires (figure 1, Protocol). Depressive symptoms are assessed with the Geriatric Depression Scale. To measure sleep, patients complete the Athens Insomnia Scale, the Dutch Sleep Disorders Questionnaire and the Pittsburg Sleep Quality Index. A neuropsychological test battery is administered as well. The primary caregiver fills out the Zarit Burden Interview and the Self-Perceived Pressure from Informal Care.

Subsequently, in order to measure skin temperature, 9 temperature loggers are placed on the body (thighs, abdomen, soles of the hands and feet), to be worn for 24 hours. During this time patients also wear a heart rate recorder. An actiwatch is placed on the non-dominant wrist for two weeks to monitor activity rhythms and to estimate sleep. In the same period, a pressure pad connected to a data logger is placed on the patients' bed at shoulder-level, underneath their bed-sheet, to determine the period people spend in bed. The logger, which is placed beside the bed, includes a light sensor for a determination of lights out time. On the second day of the protocol, patients collect 11 saliva samples, from which the diurnal pattern of cortisol levels is determined.

All proceedings are discussed in detail below.

### Participants

Patients are recruited (1) by screening patients diagnosed or treated at the Alzheimer Center of the VU University medical center, Amsterdam, (2) referred from a number of collaborating neurologists and gerontologists in the North Holland region, (3) through reactions to an advertisement in a popular magazine for elderly (PLUS), (4) through enlisting at the "Ontmoetingcentra for Dementerenden" (Meeting Centres Support Program) in North Holland. Patients' partners and volunteers from the local community are included in the control group.

### Inclusion and exclusion criteria

All patients must be between 50 and 80 years of age. Because we aim to study the effects in the earliest stage of developing memory problems we include not only people with a clinical diagnosis of probable (presenile) Alzheimer's Disease ((pre-)AD) but also people with Mild Cognitive Impairment (MCI), diagnosed by a neurologist or gerontologist according to the DSM-IV [58] or the NINCDS-ADRDA criteria [59] and the MCI- standard set by Petersen et al (2001) [60] as well as visitors of the Alzheimer center who do not fulfill the above mentioned criteria but suffer from Subjective Memory Complaints (SMC). A Mini Mental State Exam (MMSE) score of or above 14 is required for all participants at inclusion [61]. No restrictions are made for medication use.

Patients are excluded from participation in the study if diagnosed with (1) any other neurological disorder, including narcolepsy, (2) any psychiatric disorder, with the exception of mild depressive symptoms, (3) serious problems with activities of daily living (ADL), (4) sleep apnoea or restless legs syndrome or (5) a serious eye disease incompatible with light therapy, such as aphakia or retinitis pigmentosa. Healthy controls must be free of any clinical diagnosis, and not have subjective memory complaints. They also must meet the exclusion criteria mentioned above. They are selected to match the age and gender distribution of the patients. The MMSE cutoff score is ≥ 28.

### Interventions

Light boxes are installed at the patients' home, in a room of their preference. In the active condition, light boxes provide a light intensity of ~10,000 lux measured at the eye level in the gaze direction. A valid placebo condition was created by random assignment of half of the participants to a seemingly identical light box designed to produce light levels not exceeding ± 300 lux at eye level in the gaze direction. In these light boxes, the reflecting mirrors were not placed in an optimal position, full-band filters were applied and tubes of lower intensity were used. Intensities are quantified using a lux meter.

During the two-year treatment period, participants expose themselves daily to the light in sessions lasting 30 minutes every morning and evening. During this time patients do activities of their own choice such as reading, eating, watching television or working at a computer. To limit day-to-day variation in exposure timing, yet providing some individual flexibility, patients decide on a convenient fixed time-window for both the morning and evening sessions. They are free to choose their 30 minute exposures during these 90 minutes time-windows, when light is automatically switched on and cannot be switched off. If desired, lights can be switched on and off or dimmed manually at all other times of the day for use as normal lighting. The timer automatically adjusts to daylight-saving time.

### Compliance

Light therapy compliance is measured by a motion detector connected to the light box. A registration logger (DALI logger GOAL 10) is connected to a motion detector (DALI multisensor DCMC302), located on the light box for two weeks at assessment T_1 _to T_4_. Also, patients and their partners are asked at every assessment whether long periods of absence (e.g. holidays) or other compliance issues (e.g. patient being unable to remain seated for 30 minutes or illness) have occurred.

### Sample size

Under the assumption of four successful follow-up assessments and a within-subject correlation of r = 0.40, 72 participants in total would yield, at a two-sided alpha < 0.05, a power of 0.81 to detect a medium effect size of d = 0.35 for main effects [62], i.e. a difference of 0.35 of the pooled standard deviation.

### Randomization

Randomization and installation of the lights is performed by a technician not involved in the study (M. Oomstee, Netherlands Institute for Neuroscience, Amsterdam) and kept concealed. Random assignments are generated using Excel spreadsheet software (Microsoft Corporation, Redmont, USA) Codes are revealed to the researchers only after completion of the study and subsequent data reduction and processing steps.

### Statistical analysis

Prior to analyses, an automated procedure implemented in the Excel spreadsheet software (Microsoft Corporation, Redmont, USA) will be used to screen for, and exclude, extreme outliers in the outcome measure value. Excluded are observations that are at least three interquartile ranges lower than the first quartile or higher than the third quartile. Second, within-subject extremely outlying temporary changes that last only one observation are excluded. To do so, for all changes, the minimal difference to the previous or next observed value is calculated, and the distribution of these minimal differences is evaluated. Excluded are observations for which this minimal difference is three interquartile ranges lower than the first quartile or higher than the third quartile.

Mixed effect regression analysis [62] is the analysis of choice for realistic long-term data sets in psychiatry and elderly chronic care populations with variable number of available observations due to dropout or missing observations [63,64]. The analyses are performed with the MLwiN software (version 2.0, Institute of Education, London, UK) and account for the two-level nested structure of the dataset, i.e. a variable number of observations of five or less nested within participants. Light is dummy coded indicating the assignment to active treatment. For the primary outcome measure and other variables without expected gradually increasing response over time, a second dummy codes whether the observation is prior to or during treatment. Analyses apply the full factorial design with the factors light assignment group, post vs. pre assessment, and their interaction. The latter effect is the effect of interest. For secondary outcome measures with an expected gradually increasing response change over time, both pre-post treatment-effects (i.e. independent of time) and time-by-treatment effects (i.e., treatment effects evolving slowly over time) are evaluated. In ancillary analyses, the regression models allowed for evaluation of linear changes over time, and for modification of level, time course and treatment effect by missing data patterns. Logistic mixed effect regression is applied in order to evaluate possible pre-post group differences and group by time interactions in binary outcomes such as the prescription of medication.

### Approval

This study follows the Helsinki Declaration's principles, meaning that all patients sign a written informed consent stating that participation is voluntary and that participation can be withdrawn at any time, without any negative consequences concerning their current or future medical treatment. Approval has been obtained from the medical ethical committee of the VU University medical center (Protocol 2005/10).

### Primary Outcome measure

Depressive symptom severity during T_1 _to T_4 _relative to T_0 _as assessed using the 30-item version of the Geriatric Depression Scale (GDS).

### Secondary Outcome Measures

Secondary outcome measures are (1) sleep efficiency and sleep-wake rhythm amplitude and variability as estimated from actigraphy; (2) cognitive performance as assessed by the NTB (3) caregiver burden as assessed using the questionnaires Zarit Burden Interview and Self-Perceived Pressure from Informal Care. All contrasts compare T_1 _to T_4 _relative to T_0_. In order to obtain insight in underlying mechanisms, the study will evaluate whether the effects of light on mood and cognition are in part mediated by its effect on the circadian pacemaker, as read out from the rhythms in activity, body temperature and cortisol.

### Adverse effects

At each assessment patients are asked to report any side effects. Also, between three to six weeks after the light box installment, patients are contacted by telephone to ask them about any acute negative effects.

After finishing the study, or when stopping prematurely, all patients and partners received an evaluation form including a screening for adverse effects (shaking hands, stomach cramps, sweating, hunger, weakness, drowsiness, constipation, dizziness, 'sleeping' limps, nausea, irritability, hyperactivity, inability to sleep, headache and irritation to the eyes) rated by a visual analog scale.

### Cortisol measures

The saliva cortisol profile during wake is obtained at all assessments. The sampling protocol captures the morning cortisol awakening response by four saliva samples at wake up time, plus 20 minutes, plus 40 minutes and plus 60 minutes. If patients wake up before 07:00, an additional collection is made at 08:00. The pre-sleep levels are assessed at bedtime, minus 1 hour, minus 2 hours, minus 3 hours and minus 4 hours. Additional samples are collected at 12:00, 16:00 and, if not already sampled for the pre-sleep levels, at 20:00. Saliva is sampled using Salivettes (Sarstedt AG & Co, Nümbrecht, Germany), which are stored in the patient's freezer until sent back to the investigator. Transportation via mail is a convenient way of returning material, as patients live all over the Netherlands and cortisol levels are not influenced significantly by some days outside the freezer [65,66]. Patients are instructed to rinse their mouth with water and to remain seated while collecting. No eating or drinking is allowed within twenty minutes adjacent to sampling and patients are advised to refrain from eating chocolate, bananas and eggs and from drinking caffeinated coffee or black tea on the day of sampling. No restriction is placed on smoking.

At the lab, samples are stored at - 20°C until all samples can be analyzed simultaneously [67], using the Spectria Cortisol RIA (Orion Diagnistica Oy, Espoo, Finland).

### Actigraphy and bedtimes

Sleep and activity rhythms are quantified using the Actiwatch (CamNtech Ltd., Cambridge, UK) [68], which patients wear on their non-dominant wrist for two weeks [69]. During this time, to determine bedtimes, patients sleep on a pressure sensitive pad consisting of two conductive layers separated by a perforated thin layer of insulating foam, all covered in vinyl (317-140, RS-components, Haarlem, The Netherlands) [70]. When pressure is applied to the pad, the two conductive layers make contact through the pores of the insulating layer, providing a switch. The pressure pad is connected to a Hobo U12-06 logger with a Light Dependent Resistor (LDR, NSL-4962, Silonex, Montreal, Canada) fixed on it. Patients also keep a sleep log, registering bedtimes, lights-out times, whether partners keep the light on, wake up and get up times and any daytime naps.

Sleep parameters are obtained using the Actiware, version 5.57.0006 (Respironics, Inc, Murrysville, USA). Bedtimes extracted from the pressure pad data are implemented in the analysis and the software automatically calculates sleep efficiency (SE), total sleep duration (TST), nocturnal restlessness, average durations of nocturnal awakenings, and of uninterrupted sleep.

Several aspects of the diurnal activity rhythms are calculated as previously described [71,72]: the constancy of the 24-hour sleep-wake pattern over days called interdaily stability (IS); the rhythm fragmentation called intradaily variability (IV); the hourly average of minutes with activity during the 5 minimally active hours (L5); the hourly average of minutes with activity during the 10 most active hours (M10); and the absolute amplitude measure (AMP) of the rhythm calculated as the difference between M10 and L5. The relative amplitude (RA) is calculated by dividing AMP by the sum of L5 and M10.

### Temperature

Skin Temperature (Ts) is assessed using iButtons (type DS1921H, Maxim Integrated Products, Inc., Sunnyvale, USA), located at 9 positions (hands, feet, clavicles, abdomen and thighs) for 24 hours, with a recording interval of one minute [73]. From these 9 positions Ts_proximal _(abdomen, clavicles and thighs) and Ts_distal _(hands and feet) are calculated [74]. Averages are calculated over the out-of-bed and in-bed period, determined with the pressure pad data described above. Patients also wear a heart rate (HR) recorder (Actiheart, CamNtech Ltd., Cambridge, UK) for 24 hr. HR variability can give an approximation of heat production from heart rate as well as a possibility to investigate the sympathetic to parasympathetic balance.

In addition, the environmental temperature of the bedroom and living room is measured with iButtons (type DS1922L, Maxim Integrated Products, Inc., Sunnyvale, USA) for 14 days with a 10 minute interval at one assessment (T_2 _or T_4_). Outdoor environmental temperatures during all assessments are downloaded from the Royal Dutch Meteorological Institute website http://www.knmi.nl.

### Neuropsychological tests

At all assessments T_0 _to T_4_, patients complete the Mini Mental State Examination (MMSE) [61] and a neuropsychological test battery (NTB), aimed at covering a wide range of cognitive domains: short and long term verbal memory (the 15 Word List, [75]; the MIS+, developed in-house at the VUmc Alzheimer Center, no published norm values); semantic memory (WAIS Information from the Wechsler Adult Intelligence Scale (WAIS III) [76]); working memory (Number Sequences, [76]); visual memory (the Visual Association Test (VAT) [77]). Furthermore, patients are tested for interference (The Stroop Color/word Test [78], verbal fluency (Category Fluency [79]), attention and planning (The Trailmaking Test A and B [80]).

Additionally, at T_0 _visual memory is more extensively tested (Rey Complex Figure [81]; WAIS III subtest Symbol Substitution [76]); as well as motor perseveration (Amsterdam Dementia Screening subtest Meander [82]); planning (WAIS Revised subtest Maze (simplified) [83]) and flexibility and working memory (the Rule Shift subtest from the Behavior Assessment for Dysexecutive Symptoms (BADS) [84]).

### Questionnaires

All questionnaires are presented in Dutch and completed at home.

The primary outcome of depression is measured with the Geriatric Depression Scale (GDS), using the complete 30 items version [85]. The GDS is a list of statements and patients are asked to rate whether these statements are applicable to them during the last week, answering 'yes' or 'no'. The range of the cumulative score is 0 to 30 and scores are labeled as follows: 0-9 as "not depressed", 10-19 as "mildly depressed", and 20-30 as "severely depressed".

The Athens Insomnia Scale (AIS) is a questionnaire consisting of eight items, based on the criteria of the 10th edition of the International Classification of Diseases, (ICD-10) [86]. The total score ranges from 0 (absence of any sleep-related problem) to 24 (severe degree of insomnia). The responders are requested to rate an item as positive (i.e. to choose among rating options 1, 2 and 3) only if they have experienced any sleep difficulties at least three times a week during the last month. This frequency is consistent with the ICD-10 criteria for insomnia. The AIS has a cut off score of ≥ 5, the higher scores being suggestive of a diagnosis of insomnia.

The Pittsburg Sleep Quality Index (PSQI) is a 19-item scale assessing overall sleep quality over a 1-month period, with scores ranging from 0 to 21 (higher scores indicating worse sleep quality) [87]. It scores subjective sleep quality, sleep latency, sleep duration, habitual sleep efficiency, sleep disturbances, use of sleeping medication, and daytime dysfunction. A score of ≥ 5 identifies clinically significant sleep complaints.

The Dutch Sleep Disorders Questionnaire (SDQ, [88] is a questionnaire with 75 questions which can be graded by five categories ranging from 'never' to 'very often or always'. The questionnaire is derived from the Sleep Diagnostic Questionnaire by Douglass et al [89]. The questions cover 6 dimensions of sleep(-related) disorders: insomnia, periodic limb movement syndrome, excessive daytime sleepiness, narcolepsy, psychiatry, and sleep apnea syndrome. For each dimension, a subscale score can be calculated from the relevant items in the questionnaire, ranging from 1 to 5. For all dimensions the cut-off score of ≥ 3 indicate a clinically sleep(-related) problem.

'Ervaren Druk door Informele Zorg' (Self-Perceived Pressure from Informal Care, EDIZ) consists of 9 items which form a one-dimensional hierarchical scale, ranging from low to high caregiver burden [90]. Each statement can be answered with 'Yes!' 'Yes' 'More or less' (1 point), 'No', or 'No!' (0 points) creating a total score ranging from 0 to 9. A score of ≥ 4 indicates that burden is present. There are no published norm values for this scale.

The Zarit Burden Interview (ZBI) assesses the level of burden experienced by the principal caregiver of patients with dementia [91]. The principal caregiver classifies 12 propositions into four grades, ranging from 'never' to 'very much'. This yields a score with a maximum of 48 points, divided into categories where 0-10 points imply little or no burden, 11-20 points entails mild to moderate burden, 21-30 points indicate moderate to severe burden and 31-48 points means severe burden.

## List of abbreviations

AD: Alzheimer Disease; AIS: Athens Insomnia Scale; ADL: Activities of Daily Living; AMP: Absolute Amplitude Measure; BLT: Bright light therapy; DSM-IV: Diagnostic and Statistical Manual of Mental Disorders, 4th Edition; EDIZ: Ervaren Druk door Informele Zorg (Self-Perceived Pressure from Informal Care); GDS: Geriatric Depression Scale; HPA-axis: hypothalamus-pituitary adrenocortical axis; HR: heart rate; IS: Interdaily Stability; IV: Intradaily Variability: L5: 5 hours of lowest activity; M10: 10 hours of highest activity; NINCDS-ADRDA: National Institute of Neurological and Communicative Disorders and Stroke and the Alzheimer's Disease and Related Disorders Association (now known as the Alzheimer's Association); PSQI: Pittsburg Sleep Quality Inventory; RA: Relative Amplitude; SAD: seasonal affective disorder; SDL: Sleep Diagnose List; SE: Sleep efficiency; SCN: Suprachiasmatic nucleus; TST: total sleep time; ZBT: Zarit Burden Interview.

## Competing interests

The authors declare that they have no competing interests.

## Authors' contributions

EM participated in the study design, patient recruitment and trial coordination, and drafted the manuscript.

PS participated in the study design, patient recruitment and trial coordination.

EJWS is the principal investigator and initiator of the study, obtained funding, designed the study and supervised and participated in writing the manuscript. All authors have read and approved the final manuscript.
